# GMOs in Russia: Research, Society and Legislation

**Published:** 2016

**Authors:** I. V. Korobko, P. G. Georgiev, K. G. Skryabin, M. P. Kirpichnikov

**Affiliations:** Institute of Gene Biology RAS, Vavilova Str. 34/5, 119334, Moscow, Russia; The Federal Research Centre “Fundamentals of Biotechnology” RAS, Leninskii prospect Str. 33, build. 2, 119071, Moscow, Russia; Moscow State University, Leninskie gory 1, 119991, Moscow, Russia

## Abstract

Russian legislation lags behind the rapid developments witnessed in genetic engineering. Only a scientifically based and well-substantiated policy on the place of organisms that are created with the use of genetic engineering technologies and an assessment of the risks associated with them could guarantee that the breakthroughs achieved in modern genetic engineering technologies are effectively put to use in the real economy. A lack of demand for such breakthroughs in the practical field will lead to stagnation in scientific research and to a loss of expertise.

## INTRODUCTION

The history of mankind is closely linked to the selection of plants and animals in an effort to reinforce favorable traits for practical use. With scientific progress, the methods of selection have been fine tuned for an expedited generation and selection of varieties with the desired traits. The arrival of genetic engineering techniques marked another milestone in the field, representing a major breakthrough from a selection among random genetic changes to the targeted generation of organisms with the desired traits through a pre-designed modification of their genomes. Targeted genome editing technologies, besides enabling the highly efficient generation of organisms with the desired characteristics, opened up the possibility of producing foreign for organism metabolites and proteins for application in various fields, including the pharmaceutical and food industries, veterinary medicine and agriculture, as well as biotechnology and environmental protection.

The importance of genetically modified organisms (GMOs) cannot be overemphasized, as exemplified by modern pharmaceuticals, in particular recombinant proteins and vaccines, as well as by the increased efficiency in agriculture that has contributed to the drive to solve the problem of food supply, etc. Genetically modified (GM) animals are carving a place for themselves in biotechnology: in particular, as bioreactors for recombinant protein production [1]. Along with industrial use, GMOs are also invaluable tools in scientific research, from gene function studies to serving as models of human diseases. Overall, the role of GMOs in our modern world continues to grow. Meanwhile, the increasing importance of GMOs in human life and the development of targeted genome editing technologies requires that we develop well-coordinated approaches to the handling and usage of GMOs and GMO-derived products (*i.e.*, products containing or produced with the aim of or using GMOs) (GM products). Such approaches should ensure not only an optimal use of GMOs from the social and economic standpoint, but also safety in their handling.

It is important to note that circulation in the real economy of GMOs and GM products and the demand for them are closely linked to fundamental and applied research focused on the development of novel, targeted genome editing technologies and the optimization of existing ones: under conditions of a lack of demand for GMOs or prohibitive GMO turnover legislation, research in the field becomes irrelevant and atrophies. This, in turn, reinforces the dependence of transgenic research and research in targeted genome editing on the legislative framework that regulates GMO turnover and state policy in this regard. The development of biotechnology is a priority for the Russian Federation, as stated in “The Program of Development of Biotechnologies Through 2020” approved by the Government of the Russian Federation in 2012, and the companion roadmap “Development of Biotechnology and Genetic Engineering.” Genetic engineering is also in focus in the roadmap, which includes measures aiming at eliminating current inconsistencies in GMO regulation, to improve GMO related risk assessment, and to introduce cutting-edge techniques for GMO generation. Put together, these measures should sustain progress in genetic engineering, both in fundamental research and through vigorous demand in the applied sector. Owing to the initiatives contained in the Program, in the Russian Federation attention is given today to genetic engineering through the funding of research in key areas of bioeconomics in the form of programs of fundamental research, federal target programs, grants, etc. For example, in the framework of the state project “The Development of Biotechnologies and Industrial Adaptation of High-Reproduction Agricultural Plant GM Seed Production,” the first transgenic (*Bt*) potato varieties were developed in Russia, including the resistant-to-the-coloradopotato- beetle varieties Elizaveta Plus and Lugovskoi Plus. The benefits of the Russian *Bt*-potato lines are their stability, cost-efficiency, easy cultivation, and the environmental benefits of not needing insecticides. The two potato varieties have been approved for marketing by the Government (2005 and 2006, respectively). The varieties are listed in the State Registry of Varieties and Selection Achievements (2009) and covered by patents of the Russian Federation [2, 3, 4, 5, 6, 7, 8, 9, 10]. In 2015, The Russian Science Foundation (RSF) announced a call for research proposals that addressed various research priorities, among which was the development of techniques for the production of pharmaceuticals in eukaryotic systems, including plants and animals as bioreactors. Following a review of the applications, three proposals received financial support, which, along with other goals, aimed to develop novel approaches in animal transgenesis on the basis of existing best practices in the field. This will make it possible not just to generate animals producing recombinant proteins for practical use, firstly in medicine and pharmaceutical applications, but also improve the safety of the recombinant proteins and minimize the potential risks to consumers associated with them. These examples clearly indicate an orientation of the state’s policy towards both preserving and building up expertise in this field. However, in contrast to the priorities set forth in the Complex Program and the Roadmap, the current legislative framework does not support the practical use of GM animals and plants, whereas the anticipated changes in it and the proposed solutions have some significant drawbacks. If ignored, this situation will not encourage the adoption of practical decisions in this sector of the economy, which is at the moment characterized by legislative uncertainty and anemic growth, while it is an innovative and hi-tech sector.

## THE GMO LEGISLATIVE FRAMEWORK IN THE RUSSIAN FEDERATION

As of today, circulation of GMOs in the Russian Federation is regulated by Federal Law dated June 05, 1996, No. 86-FZ (edit. on June 19, 2011) “On the State Regulation in Genetic Engineering” (hereafter “86-FZ”) and by Government Decree of the Russian Federation dated February 16, 2001, No. 120 “On the State Registration of Genetically Modified Organisms”. In addition, turnover of GMOs for some particular use, such as food, feeds, and feed supplements, is regulated by national normative documents or recent acts of the Customs Union, such as Customs Union Technical Regulations TR CU 021/2011, TR CU 022/2011, TR CU 027/2012, TR CU 029/2012. However, mechanisms of state registration of GMOs intended for release into the environment, bylaws, and normative documents guiding the state registration process are lacking. Under these conditions, cultivation and breeding of GMOs, for example in agriculture, is ruled out. Furthermore, since the adoption of 86-FZ, genetic engineering techniques have undergone significant progress. The methods currently used allow for targeted genome editing without leaving foreign DNA sequences behind, collectively known as scarless genome editing, such as CRISPR (Clustered Regularly Interspaced Short Palindromic Repeats), TALEN (Transcription Activator- Like Effector Nucleases), and ZFN (Zinc Finger Nucleases) [11, 12, 13, 14]. Also, there is a trend away from first- and second-generation GM plants, which are generated through the introduction of foreign DNA sequences into a recipient organism, toward third- and fourth-generation GMOs that lack foreign genetic material in their genomes. These trends definitely require an unambiguous legislative definition and positioning. This situation, which *de facto *makes impossible state registration of GMOs and the obtaining of approval for field farming, is supposed to be addressed by Government Decree of September, 2013, No. 839 “On the State Registration of Genetically Modified Organisms Intended for Release into the Environment, As Well As Products Obtained with the Use of Such Organisms or Containing Such Organisms” (hereinafter Decree No. 839), slated to enter into force on July 01, 2017. Decree No. 839 regulates the procedures of state registration and approval for a permitted use of GMOs intended for release into the environment, as well as products containing or produced with the use of such organisms. Yet, Decree No. 839 nullifies Decree No. 120 of the Russian Federation dated February 16, 2001. Decree No. 839 differentiates GMOs based on their intended use, subject to the implementation of procedures developed by a corresponding body of executive power for conducting assessments suitable for each type of intended use. It is now clear that the list of intended uses of GMOs stated in Decree No. 839 (manufacturing of human and veterinary pharmaceuticals, medical devices, food, feeds and feed supplements, breeding and/or cultivation on the territory of the Russian Federation of GM plants, animals, and agricultural microorganisms) is far from comprehensive, thus potentially raising hurdles in the future for GMO use in certain applications. Namely, today several field trials of GM mosquitoes designed to eliminate mosquito-transmitted human diseases, such as Denge fever, are underway [15, 16, 17]. Clearly, neither of the types of GMO intended use listed in Decree No. 839 covers this example, which can be defined as “environmental modification.” Also, Decree No. 839 does not specify the possibility of registration of GMOs and GMO products in such a dynamically developing and economically important sector as “technical use,” such as biofuel production, GM cotton, etc.

Decree No. 839 implies that state registration of GMOs is contingent on issuing a permit for its intended use. In other words, if an application for a permit is rejected, there is no state registration of the GMO and the GMO does not get listed in the state registry. At the same time, we believe that one of the vital objectives in the regulations of GMO turnover in the Russian Federation is the collection of information on GMOs that hold potential for practical use (even despite the absence of a permit for use), which would allow for their identification and, if required, monitoring. In the case of a lack of GMO recordkeeping, irrelevant of the issuing of a permit for use, there is a risk of their illegal use with no technical capability for their identification and revealing facts of unauthorized use. In this regard, it is possible to introduce GMO recordkeeping (for example, in the form of a consolidated GMO registry) which would be independent of the outcome of the state registration process and supposed accumulation of data on GMOs, their genetic modification, and methods of identification and monitoring.

A possible drawback of the state registration process in its current form is the execution of an expertise of GMO molecular genetic study results by several federal bodies, depending on the type of intended use. Therefore, depending on the type of intended use of a GMO and the body of executive power responsible for its registration, the required experimental data and proofs might vary. There is no doubt that the workload in testing and the type of laboratory assays should account for GMO type specifics, details of its exploitation, and the intended use. However, it would be rational to harmonize and standardize molecular genetic characterization independently of the intended use of a GMO, and to identify molecular genetic expertise as a unified step that presupposes the deposition of information on the GMO into the united GMO registry within the procedure of GMO state registration, notwithstanding whether a permit for use is granted or not.

Finally, Decree No. 839 addresses only the question of state registration of GMOs intended for release into the environment. At the same time, Decree No. 839 (as well as TR CU 021/2011 “On the Safety of Food Products”) requires state registration of a GMO as a mandatory condition for the registration of products obtained with the use of that GMO, regardless of whether the GMO is released into the open environment or used in a closed system (*i.e.*, not assuming contact of the GMO with the environment). Therefore, GM products derived from GMOs grown and bred in a closed system without a release into the environment cannot be registered due to the lack of regulations covering the registration of GMOs not intended for release into the environment. Thus, there is now an objective need for a legal basis for state registration of GMOs used for production purposes which are not intended for release into the environment.

The third milestone in GMO legislation, besides FZ-86 and Decree No. 839, is the recently introduced Federal Law of July 3, 2016 No. 358-FZ “On the Amendments to Individual Legislative Acts of the Russian Federation Improving State Regulation in Genetic Engineering” (hereafter –358-FZ). These amendments prohibit the cultivation and breeding of GM plants and animals, except for research and laboratory purposes. Importantly, Federal Law 358 only bans GM animals and plants “whose genetic program has been altered using genetic engineering methods and containing genetically engineered material whose appearance cannot be the result of natural processes” (358-FZ, Article 4). Therefore, third- and fourthgeneration GMOs, whose genome alteration theoretically can occur naturally without genetic engineering intervention, are exempt from the prohibition, which requires a greater effort to adopt an unambiguous legal definition of such organisms and products containing such organisms or obtained with the use of such organisms.

Despite the fact that some organisms generated with the use of genetic engineering are not banned by 358-FZ, prohibitive measures might have a negative impact on this sector of the economy, which is one of the drivers of innovation. The current situation is made worse by a lack of prohibitive measures for GM products, along with the ban on the cultivation and breeding of GM plants and animals. In a case of a need for a GM plant- or animal-derived product, there is a risk of becoming fully dependent on external sources of GMOs.

The current ban on the cultivation and breeding of GM plants and animals could also affect research in the field of plant and animal transgenesis (and, accordingly, their financial support), both fundamental and translational ones. In such a situation, the Russian Federation can quickly loose its position and expertise in this area, finally falling into a dependence on external sources of GMO supply. In particular, should the ban be adopted, it would make impossible GM animal-derived pharmaceutical (and other) recombinant protein production from milk, which, according to the RAND Corp., a highly authoritative analytical entity, will become one of the leading trends in biotechnology, bionanotechnology and biomedicine to 2020 [18]. This opinion is supported by the presence on the market of the ATryn® and Ruconest ® pharmaceuticals, which are based on the recombinant human antithrombin III and C1-esterase inhibitors, which are derived from GM goat and rabbit milk, respectively [1].

## WHAT IS A GMO?

The term “GMO” is the cornerstone of the field, since it defines the subject of regulation. As of today, Federal Law 86-FZ defines a GMO as “an organism or several organisms, any non-cellular, cellular and multicellular formation capable of reproduction or transmission of its own genetic material, different from wild-type [natural] organisms, generated using genetic engineering methods and carrying genetically engineered material, including genes, their fragments, or combinations of genes.” On the one hand, this definition is very broad, and, based on it, plasmids, actually being vectors whose propagation is possible only in permissive host cells, also fall under this definition. On the other hand, the requirement of reproducing and transmitting genetic material exempts infertile GMOs, such as the hybrids of fertile GMOs. At the same time, the transfer of genetic modification on a novel genetic background occurring as a result of crossing can affect its manifestations and requires a separate assessment of the risks and safety aspects. For this reason, the definition of a GMO should also cover such organisms. Finally, with the arrival of scareless genome editing technologies not assuming the introduction of foreign genetic material, a legal status for organisms obtained with the use of such technologies should be defined. Such organisms, classified as third- and fourth-generation GMOs (see section “GMO classification”), are indeed products of genetic engineering. However, they can in theory appear through natural selection, thus meaning full principal identity of a genetic engineering manipulation to natural processes in this case, and a scientifically substantiated classification of such organisms as being non-GMOs. Along with that, because the genome of such organisms carries no scarring sequences, the GM origin of such organisms is impossible to objectively prove, unlike in the case of “classical” GMOs of the first and second generations, which bear foreign DNA in their genomes serving as proof of their genetic modification. Evidently, the impossibility of proving usage of genetic engineering methods in the generation of such organisms can lead to legal ambiguity. In light of the abovementioned concerns, it appears rational to lump organisms with genetically engineered genomes but lacking foreign DNA with those obtained through a classical selection process, while the notion of GMO should be restricted to those bearing in their genetic material foreign DNA sequences. This view is also shared by the international scientific community [19].

To summarize, there is currently a need for amendments to the definition of “a genetically modified organism,” which should, on the one hand, address the shortcomings of the existing definition (both its redundancy and insufficiency) illustrated above, and on the other, unambiguously situate in the legal realm organisms generated with the use of scarless genome editing methods, which have the potential to appear as a result of natural processes. Federal Law 358-FZ which exempts genetically engineered organisms in which genetic modifications can result from natural processes implicitly treats such organisms as outside the GMO classification, which, however, must be evidently specified in normative acts. As alluded to above, GMOs could be defined as “non-cellular, single-cellular or multicellular formations generated with the use of genetic engineering and containing foreign DNA sequences that cannot appear as a result of natural mating and horizontal gene transfer between non-GMO organisms, and/or as a result of recombination events or mutations (deletions and insertion of endogenous genetic material, single nucleotide substitutions, chromosome rearrangements).” Other organisms generated with the use of genetic engineering technologies shall not be considered as GMOs.

## THE SAFETY OF GMOS

One of the obstacles in the use of GMOs is the concerns related to their safety. The safety of GMOs and GM-products can be divided into the safety for consumers and environmental safety.

Today, there is a belief, mainly related to food products, that the presence of a genetic modification a priori makes GM products dangerous for humans. However, no scientific study has revealed negative effects related to the consumption of GM food products ostensibly caused by the presence of a genetic modification per se. As for the published results of studies apparently demonstrating the side effects related to GMO consumption and which are used as reference, a detailed analysis of such studies reveals scientific and methodological flaws and, consequently, shaky findings [20]. Indeed, the presence of foreign DNA in a host’s genome contained in a food product by no means affects the safety of such a product for consumers: (i) first, the lack of horizontal DNA transfer upon ingestion has been experimentally demonstrated for mammals [21]; (ii) second, human diets contain huge amounts of foreign DNA from plants and animals and no horizontal transfer has yet been documented.

Interestingly, GMOs have constituted a portion of our diet for the last several thousand years according to recent studies. Namely, the genome of the sweet potato plant domesticated approximately 8,000 years ago has been shown to contain two stretches of DNA derived from the genome of *Agrobacterium*, one of which, in the authors’ opinion, conferred the desirable traits that warranted the domestication of this particular variety [22]. At the same time, *Agrobacterium *T-DNAbased vectors are widely used today in plant genetic engineering [23]. Overall, the consumption of naturally transgenic sweet potato for a thousand years demonstrates that transgenic food crops are safe for humans from the dietary perspective, which is a very sensitive subject in society.

Put together, the only threat posed by a product obtained with the use of a GMO or containing a GMO is the new characteristics that the new phenotype might possess. However, any risk assessment should be based on our common regulations for new non- GMO products, which carry risks just as well [24]. The potato variety Lenape, which was withdrawn from the market due to excessive accumulation of natural potato toxins eventually formed during random selection is a good example of the risks associated with organisms obtained through natural selection [25].

In summary, the risks related to genetic modifications *per se *are negligible, whereas organisms obtained without the use of genetic engineering, similarly to GMOs, may also be unsafe.

## CLASSIFICATION OF GMO

GMO classification is of practical importance since monitoring strategies and risk assessments studies of GMOs and GM products should be guided by their intrinsic characteristics.

One of the common classification schemes is by generation. It has been historically used for GM plants (and is fully applicable to GM animals). The first-generation GMO group includes organisms that carry a fragment of exogenous DNA in their genome. The organisms that belong to the second- generation GMOs are similar to those of the first group but carry several transformation events and can be obtained by crossing first-generation GMOs. Owing to the presence of foreign DNA in the genome of first- and second-generation GMOs, these organisms can be unambiguously identified [26], and scarring sequences unequivocally provide evidence of past genetic manipulations. Third- and fourth-generation GMOs, which are nearly intragenic (i.e., carry endogenous DNA sequences with minimal modifications), intragenic and cis-genic organisms (modified essentially with authentic endogenous genetic material), should be treated differently from the abovementioned GMOs [26]. The prominent feature of these organisms is the defining possibility of natural occurrence of such genetic medications in the wild or during selection through mutations and/ or chromosome rearrangements. This, in turn, leads to the impossibility of proving objectively that genetic engineering has been used to generate such an organism. Having said that, it would seem logical that third- and fourth-generation GMOs should be treated within the legal framework as those obtained through a classical selection process.

For GMOs ascribed to the first and second generations, safety and risk assessments and applicable constraints are mostly identical, with the requirement for more than one transformation event analysis for second-generation GMO identification and monitoring. In our opinion, from a practical point of view, more important in GMO and GM product classification are the following criteria:

•cultivation and breeding in a closed system (i.e., assuming no contact with the environment) or in an open environment;

•presence or absence of viable or inactivated GMOs in GMO-derived products; and

•presence or absence of GMO-derived DNA in GM products.

The abovementioned criteria allow for shaping optimal and well calibrated principles of both GMO characterization from the molecular genetics standpoint and risk assessment for GMOs and GM products.

## APPROACHES TO THE RISK ASSESSMENT OF GMO AND GM PRODUCTS

**GMO indented for cultivation and breeding in a closed system**

A GMO of this type primarily has no contact with the environment, while contacts with humans are restricted to the manufacturing process this organism is used in, and the personnel involved in GMO processing. In this respect, there is a no need for an assessment of the impact of GMOs on the environment and of the related risks, which might exist only for manufacture waste. The potential risks to personnel during GMO handling are comparable to those faced when handling similar non-GMOs, with additional requirements to assess the risks linked to the presence of genetic modifications. Keeping in mind that GMOs may spill into the environment in an emergency situation, there should be pre-established strategies for GMO identification and monitoring based on unique transgene detection, as well as measures to eliminate the consequences of a release into the environment. At the same time, the necessity for a molecular characterization of the transformation event in a given GMO (a transformation event refers to the incorporation of an exogenous DNA fragment into a particular site of an organism’s genome) should depend on the type of GMO-derived product (see below).

**GMO intended for cultivation and breeding in an open environment**

This type of GMO requires that the risks be assessed in terms of the interactions of such organisms with the environment and the potential impact on it. These risks can be divided into two groups. The first is related to the novel phenotypic features acquired by an organism as a result of a genetic modification, as well as to the intrinsic properties of the recipient organism if introduced into an extrinsic ecosystem. The second relates to the risk of uncontrolled propagation of the genetic modification in the ecosystem. To evaluate the first group of risks, it would be rational to implicate the approaches, methods, and criteria used in the evaluation when a similar non-GMO is introduced into the ecosystem as a novel species. Such an evaluation could also incorporate an assessment of the risks arising from the production by the GMO of extrinsic proteins and metabolites as a result of the genetic modification. However, as of today, ecological expertise of novel breeds and varieties is not stipulated by the law, while experience in such types of expertise applicable to cases of novel species introduction is very limited. This lack of appropriate knowledge and experience hinders an efficient application of such an approach and needs to be remedied by scientifically proven guidelines to assess the environmental impact of GMOs, which would also be fully applicable to non-GMOs. Indeed, regardless of whether the resistance of an organism to environmental factors (for example, to a particular pathogen) was conferred by a genetic manipulation or occurred naturally, the risks associated with the release of such an organism into the environment are similar, and the assessment of the environmental impact of an introduction should be done for both GM and non-GM organisms. In this case, it is rational to build the assessment on the basis of a comparative analysis with a similar organism (for GMO – with the recipient organism).

The presence of a transgene adds additional requirements to the risk assessment strategy. This includes uncontrolled horizontal or vertical transgene transfer (importantly, the risks of a spread of traits (for example, resistance to pathogens and pests) acquired under classical selection are the same, but they are not covered by the current rules). It is possible to conduct an assessment of the risks of uncontrollable transgene expansion in the ecosystem depending on the specific properties of the GMO and type of genetic modification. For instance, GM animals show negligible risks of horizontal transgene transfer, whereas the risks associated with vertical inheritance of a transgene following inbreeding should be taken into account. In contrast, for GM microorganisms the assessment of horizontal transgene transfer is a must. In order to avoid bias, studies and methods aimed at evaluating horizontal or vertical transgene transfer risks should be standardized as much as possible for different taxonomy groups of GMOs and their intended use.

In compliance with Decree No. 839, the safety of a GM organism is the only factor that affects the decision on the release of such an organism into the environment (except for GMOs intended for the production of pharmaceuticals and medical devices). At the same time, all newly acquired properties, regardless of the mechanism of acquisition,* a priori *act as risk factors due to the fact that a comprehensive analysis of the environmental impact is impossible, thus prompting an unconditional ban in order to exclude all possible risks. Having said that, the decision on the cultivation and breeding of GMOs should consider not only identified or potential environmental risks, but other factors also should be taken into account, such as technological, social, economic factors, etc., and the final decision should be based on a comprehensive multifactorial “risks versus benefits” analysis.

The strategy regarding GMOs intended for cultivation and breeding in an open environment, in particular GM plants and animals, requires not only unambiguous identification tools enabling their monitoring, but also methods allowing for an analysis of transformation events (if the latter are present). The transformation event unambiguously identifies the line of the GMO and permits its differentiation from related lines carrying the same transgene. In this case, the transformation event can also serve as a unique feature identifying the GMO.

**GMO-derived products**

It is deemed logical that GMO-derived products deserve a differential approach taking into account the specific risks associated with the described-above product types. Along with that, a general approach to safety evaluation should be based on principles applicable to similar non-GM products, with an additional evaluation of the specific risks associated with the presence of a transgene, if any.

As discussed above, we believe appropriate to single out three subtypes of GM products. The first one is defined as “products obtained with the aim of GMOs” and covers products manufactured from GMOs or their “waste products,” or the latter themselves, which are free of GMO genetic material (the maximally allowed residual DNA content should be settled in this case and controlled). Recombinant proteins and target metabolites (amino acids, etc.) are examples of such products. When compared to similar non-GM products, such GM-derived products pose no additional risks because of the absence of transgenic material. On these grounds, such products can and should be treated as non-GM. The only parameter worth monitoring is ensuring that there is no residual transgenic material in a manner similar to the regulatory standards of quality control for biopharmaceuticals, implying a maximally allowed residual host strain DNA content. For GMOs used for the manufacturing of this type of products and not supposed to be released into the environment, there is no need for transformation event description, if the latter exists.

The second type of GM products consists of “products obtained with the use of GMOs” which contain whole non-viable GMOs or products of their processing not assuming the removal of host DNA. The additional risks posed by such GM products are linked to the presence of GMO DNA and the associated potential risks of a horizontal transfer, which should govern risk assessment in conjunction with screening for viable organisms.

Finally, the third type of GM products can be defined as “products containing or being viable GMOs,” thus presupposing the presence of viable GMOs. The greater number of additional risks is associated with this type of products if compared to analogous non- GM products. In this case, an assessment of the risk of uncontrolled expansion of the GMO derived from the product in the environment should be performed, and if significant, necessitate a whole complex of risk assessment tools suited to GMOs intended for release into the environment.

**Modern technologies of targeted genome editing and GMO’s molecular genetic characterization and safety evaluation**

As noted above, the legal framework for GMO and GM product turnover could directly influence research in the field of genetic engineering. At the same time, advances in targeted genome editing technologies, along with their practical applications aiming at generating socioeconomically significant GMOs, could be of importance for GMO and GM product characterization and safety evaluation, something especially applicable to GMOs bearing a transgene integrated into the host genome.

Earlier techniques of plant and animal transgenesis resulted in random integration of transgene into the host genome at variable copy numbers. Along with significant variations in transgene expression efficiency and stability, it technically complicates the precise localization of the transgene integration site in the genome (i.e., transformation event), especially in the case of tandem integration of multiple transgene copies. In addition, random incorporation of a transgene can potentially lead to side effects that affect the safety of such organisms. For example, altered protein isoforms might appear, or some metabolic pathways could be altered, etc. Current methods of targeted genome editing, such as those based on CRISPR-mediated homologous recombination, along with its combination with site-directed recombination (using Cre, Flp and other recombinases) to increase the efficiency of transgenesis, allow one to achieve a targeted integration of a transgene with single-nucleotide precision. This approach enables the selection of an optimal integration site in the recipient’s genome. For example, the β-casein locus may be an optimal one for transgene insertion to efficiently produce recombinant proteins in milk, owing to its high endogenous expression level and the dispensability of β-casein for normal lactation [27, 28]. Other attractive loci are those capable of supporting transgenic expression upon insertion but whose integrity is indispensable for the normal growth and development of an organism, such as ROSA26 locus [29, 30, 31, 32]. Besides the efficient generation of transgenic organisms with ensured stability and efficiency of transgene expression and a minimized likelihood of eventual adverse effects on the host due to transgene insertion, which is of importance in ensuring the organism’s safety, targeted genome editing technologies allow one to control the transgene copy number and makes the description of the transformation event a routine task. Over all, the use of state-of-the art methods of targeted genome editing simplifies the essential, for state registration, molecular genetic research aimed at characterizing the generated organisms and contributes to the minimization of the risks associated with the influence of a genetic modification on the GMO safety profile compared to its non-GMO counterpart (recipient).

## CONCLUSION

To summarize, today it is vital to revisit our legal framework and guidelines related to the safety and risk assessment of GMOs and GM products in the Russian Federation. The suggested herein concept enables to conduct an efficient evaluation, while eliminating wasteful studies depending on the specific features of a GMO, the conditions of its intended handling, and the features of the derived GM product. The creation of a system that enables a broad involvement of GMOs in the real economy will also provide incentives for research in this dynamic and growing field, where the Russian Federation today has sufficient expertise and potential [33]. However, if the situation with the regulatory system remains unchanged, with a total ban on GM plants and GM animals remaining in place, the existing expertise might be rapidly lost because it won’t be needed.
